# Do Power Lines and Protected Areas Present a Catch-22 Situation for Cape Vultures (*Gyps coprotheres*)?

**DOI:** 10.1371/journal.pone.0076794

**Published:** 2013-10-09

**Authors:** W. Louis Phipps, Kerri Wolter, Michael D. Michael, Lynne M. MacTavish, Richard W. Yarnell

**Affiliations:** 1 School of Animal, Rural and Environmental Sciences, Nottingham Trent University, Southwell, Nottinghamshire, United Kingdom; 2 VulPro, Rietfontein, North West Province, South Africa; 3 Research, Testing and Development, Eskom Holdings Ltd, Johannesburg, Gauteng Province, South Africa; 4 Mankwe Wildlife Reserve, Mogwase, North West Province, South Africa; University of Marburg Germany

## Abstract

Cape vulture *Gyps coprotheres* populations have declined across their range due to multiple anthropogenic threats. Their susceptibility to fatal collisions with the expanding power line network and the prevalence of carcasses contaminated with illegal poisons and other threats outside protected areas are thought to be the primary drivers of declines in southern Africa. We used GPS-GSM units to track the movements and delineate the home ranges of five adult (mean ±SD minimum convex polygon area  =  121,655±90,845 km^2^) and four immature (mean ±SD minimum convex polygon area  =  492,300±259,427 km^2^) Cape vultures to investigate the influence of power lines and their use of protected areas. The vultures travelled more than 1,000 km from the capture site and collectively entered five different countries in southern Africa. Their movement patterns and core foraging ranges were closely associated with the spatial distribution of transmission power lines and we present evidence that the construction of power lines has allowed the species to extend its range to areas previously devoid of suitable perches. The distribution of locations of known Cape vulture mortalities caused by interactions with power lines corresponded to the core ranges of the tracked vultures. Although some of the vultures regularly roosted at breeding colonies located inside protected areas the majority of foraging activity took place on unprotected farmland. Their ability to travel vast distances very quickly and the high proportion of time they spend in the vicinity of power lines and outside protected areas make Cape vultures especially vulnerable to negative interactions with the expanding power line network and the full range of threats across the region. Co-ordinated cross-border conservation strategies beyond the protected area network will therefore be necessary to ensure the future survival of threatened vultures in Africa.

## Introduction

Vultures in the *Gyps* genus are obligate scavengers of vertebrate carcasses and provide vital ecosystem services by recycling carrion, thereby limiting the development and spread of disease and maintaining energy transfer through food webs [1], [2]. Their longevity, delayed maturity and low reproductive rates mean that even minimal reductions in adult survival rates or the proportion of immatures reaching breeding age could result in population declines [3], [4]. As a consequence all eight species of *Gyps* vultures found globally are declining [5] because of multiple threats such as reduced food availability [6], [7], illegal poisoning [8], and collisions with wind turbines [3], [9] and power lines [10]. The recent collapse of *Gyps* vulture populations in Asia caused by accidental contamination of their food supply [11] has resulted in major changes to scavenger community dynamics and a wide range of human health and socio-economic impacts in the region [12]. The urgency to prevent similar ecological catastrophes from occurring elsewhere is widely acknowledged [5], [11].

African vulture populations have also declined considerably, with land use change and illegal poisoning identified as widespread mortality factors [13]–[15]. For example, a 52% decline in *Gyps* vulture numbers in the Masai Mara region of Kenya over a 30 year period was largely attributed to secondary poisoning after they fed on carcasses illegally laced with poisons to kill livestock predators [15]. Electrocutions and collisions with the expanding power line network are also frequent causes of vulture mortality and injury in Africa [16], [17]. *Gyps* vultures are especially prone to fatal interactions with power lines in southern Africa due to their frequent use of power line towers for perching and roosting [4], [18]. For example, in the Eastern Cape Province of South Africa it is conservatively estimated that fatal interactions with power lines kill *ca.* 4% of the local population of Cape vultures *G. coprotheres* per year, with the possibility of rapid local extinctions in some high risk areas [16]. Despite this vultures might have derived some benefits from the presence of power lines. For example, African white-backed vultures *G. africanus* nest on pylons [19] and it has even been suggested that some areas previously devoid of suitable perches might become newly accessible as vultures utilise power line towers as roost sites and vantage points [4]. Under the current rate of expansion of the power line network it is important to investigate the relationship between power lines and vultures in southern Africa, particularly in a spatial context to allow mitigation measures to be implemented in key areas [10], [16].

Vultures and other raptors in Africa are thought to be increasingly restricted to protected areas where they are less exposed to multiple threats that persist in the wider landscape [13]–[15]. For example, in several African countries increasing prevalence of anthropogenic mortality factors such as illegal poisoning have led to higher vulture mortality rates and population declines outside compared to inside protected areas [14], [15], [20]. While protected areas often provide safe breeding and roosting sites for vultures [15], [21]–[23], they frequently forage far beyond protected area boundaries, leaving them exposed to numerous threats [20], [24]. The role and effectiveness of protected areas for vulture ecology and conservation remains unclear, therefore, and merits further investigation.

In this study we use Global Positioning System (GPS) telemetry techniques to provide a first insight into the size and extent of Cape vulture home ranges in relation to the network of power lines and protected areas in southern Africa. The Cape vulture is endemic to southern Africa and is listed as Vulnerable on the IUCN Red List due to declines across its range [25]. It is a gregarious cliff-nesting species with a global population estimated at 8,000–10,000 individuals (*ca.* 4,000 breeding pairs) [25]. The largest remaining breeding colonies are located in the north-eastern provinces of South Africa [4], [23], [25], [26], where increasing urbanisation and land use change has caused habitat loss, food shortages and an increasing incidence of negative vulture-power line interactions [17]. We hypothesize that frequent use of transmission line pylons as perching and roosting sites by Cape vultures will influence the extent of their home ranges and the location of their core foraging areas. We also assess the ability of GPS tracking data to identify potentially high risk areas of vulture-power line interactions in order to inform future mitigation strategies. We predict that Cape vultures regularly roost and forage outside protected areas, but expect adults to traverse smaller home ranges in closer proximity to protected breeding colonies compared to the less restricted movements of immature individuals [27].

## Methods

### Vulture Captures and GPS Tracking

**Figure 1:**
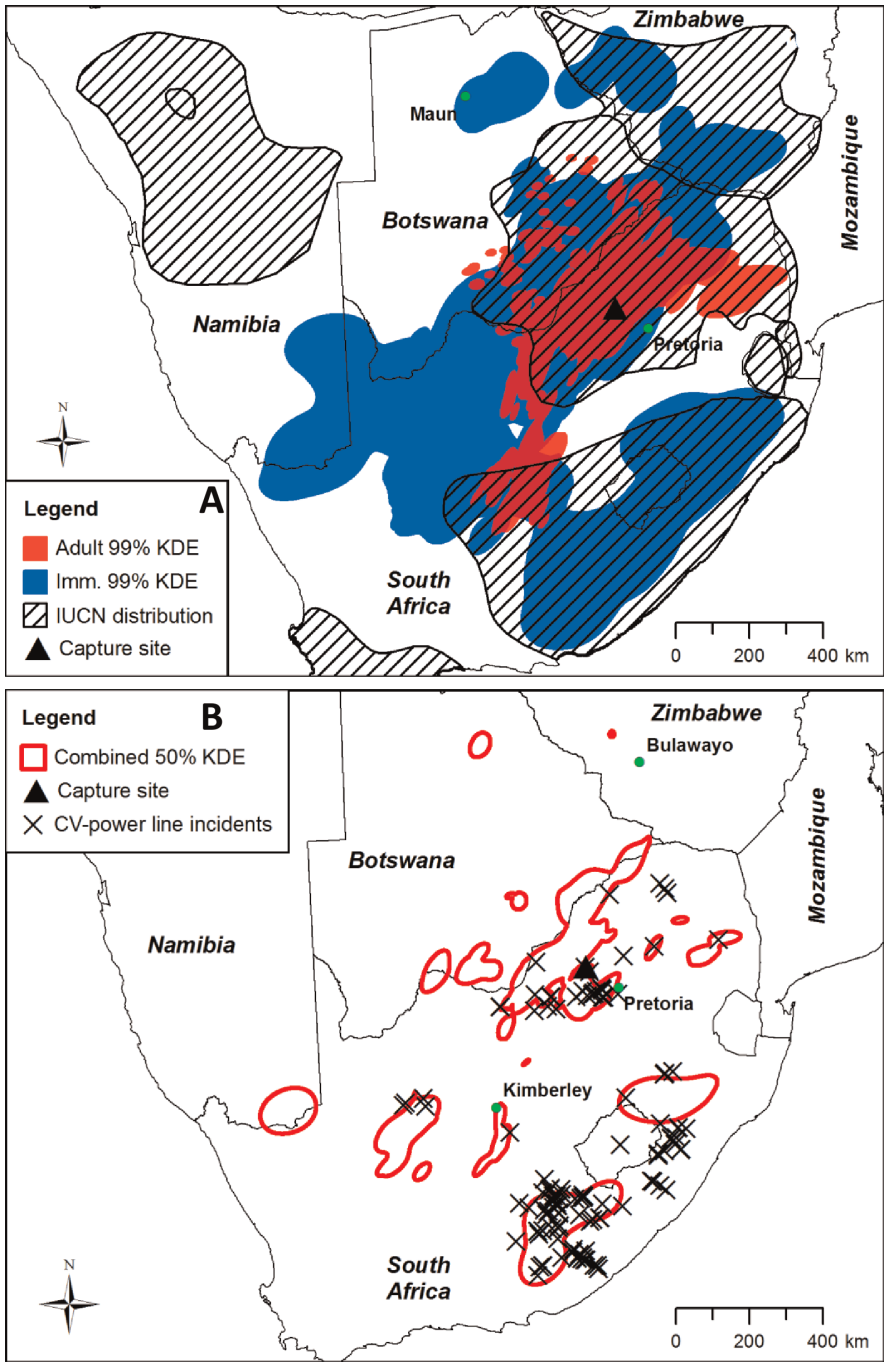
Home ranges of nine Cape vultures with species distribution map and Cape vulture-power line incidents. (A) Shaded red and dark blue polygons represent the combined 99% kernel density estimated (KDE) contours of all adult and immature vultures, respectively. The diagonal line shaded polygons represent the extent of the Cape vulture species distribution according to BirdLife International [37]. The capture site is indicated by a black triangle. (B) The hollow red polygons represent the combined 50% KDE contours of all nine vultures. Black crosses show locations of Cape vulture-power line incidents recorded in the Central Incident Register of the EWT-Eskom strategic partnership [39].

A walk-in cage trap (6×3×3 m) baited with ungulate carcasses was used to catch vultures at a supplementary feeding site for vultures at Mankwe Wildlife Reserve (MWR; 25^o^13’S, 27^ o^18’E) in the North West Province of South Africa (Fig. 1) [24]. Ten Cape vultures were caught between November 2009 and June 2010 during seven separate captures. Owing to the difficulty in ageing *Gyps* vultures individuals could only be identified as adults (>5 years), immatures (2–5 years) or juveniles (first year) based on characteristic plumage traits and eye colouration [28]. For statistical analyses juveniles (n = 1) were pooled with immatures. The genders of vultures were not confirmed by molecular analysis as blood or feather samples were not taken from each individual and *Gyps* vultures exhibit no obvious sexual dimorphism in plumage or body characteristics [29]. Furthermore, Bosé *et al.* [29] found no difference in male or female life history or movement patterns in a closely related species and so this was not investigated during this study. Teflon® ribbon backpack-style harnesses were used to secure Hawk105 GPS-GSM (Global System for Mobile communications) tracking units (Africa Wildlife Tracking Ltd., Pretoria, South Africa; www.awt.co.za) onto the back of each vulture [24], [30]. Each unit recorded GPS locations (∼10 m accuracy, verified by a positional dilution of precision (PDOP) measure of accuracy [31]), altitude above sea level, speed, direction of travel, date, time and temperature three times per day at 07:00, 11:00 and 15:00 Central Africa Time (CAT). One additional data point was recorded per day at 13:00 CAT for three adult vultures (AG314, AG349 and AG355) to account for foraging trips from cliff roosts between the 11:00 and 15:00 readings. The units were expected to record and transmit data for approximately one year. Patagial tags with unique numeric codes were attached to both wings of each vulture to allow identification of individuals following release.

### Ethics Statement

The procedures were approved by the ethical review committee of the School of Animal, Rural and Environmental Science, Nottingham Trent University. Permits for the capture and handling of vultures and the fitting of tracking units were granted by the Department of Agriculture, Conservation, Environment and Rural Development, North West Provincial Government, Republic of South Africa (Permit: 000085 NW-09). All procedures were carried out by South African Bird Ringing Unit permit holders (KW and WLP). All necessary measures were taken to minimise any potential discomfort to the birds. Each tracking unit weighed 170g which is *c.* 1.8% of the mean mass of an adult Cape vulture [4], and less than the 3% recommended for flying birds. A weak point was included on each harness to allow it to eventually fall off, releasing the tracking unit from the bird.

### Data Analysis

GPS locations were projected to the Universal Transverse Mercator (UTM) coordinate system for all spatial analyses. Stationary and moving GPS locations were defined as all those recorded with a speed < or ≥ 10 km**·**h^−1^, respectively. Distances travelled between consecutive GPS locations were calculated for each vulture. Two methods were used to delineate the home ranges of each vulture.

Firstly, Minimum Convex Polygons (MCPs) were created using the Home Range Tools extension [32] for ArcGIS® using all recorded GPS locations to allow comparisons to be made with other *Gyps* vulture tracking studies [27], [33]. Incremental area analysis was performed for each vulture by creating MCPs using sequentially added consecutive GPS locations until all locations were used to create an MCP for the total tracking period. Home range area curves were then plotted to identify whether the home range areas reached asymptotes by the end of the tracking period [33]. Secondly, fixed kernel density estimation (KDE) was carried out using the Geospatial Modelling Environment (GME) program [34] to delineate 99% and 50% contours to represent the overall and core foraging ranges, respectively [35]. The plug-in method of bandwidth selection was used following preliminary analyses that indicated that the least-squares cross-validation (LSCV) method failed to select a bandwidth due to numerous identical GPS locations and use of the reference bandwidth resulted in over-smoothed home range boundaries [36]. A 1 km^2^ cell size was used for KDE calculations. The 99% KDE contours were used instead of the 95% contours to represent the overall home ranges as the latter generally produced undersmoothed and more fragmented outer contours. The size of the 99% KDE contours and MCPs of the adult and immature vultures were compared using Mann-Whitney tests. The spatial extent of the home ranges were compared to the IUCN Cape vulture species distribution map [37] and the proportion of GPS locations recorded within the IUCN distribution were compared between adults and immatures using Mann-Whitney tests to determine whether either age class travelled beyond the known species distribution more or less frequently than the other.

The use of transmission power lines and associated stuctures for perching and roosting by vultures was estimated by calculating the proportion of stationary (i.e. < 10 km**·**h^−1^) GPS locations recorded within 50 m of transmission power lines within each vulture’s home range. Analyses were performed in ArcMap v9.3 [38] and spatial data for transmission power lines were sourced from Eskom (South Africa), the Africa Infrastructure Knowledge Program (Botswana and Zimbabwe; http://www.infrastructureafrica.org) and the Environmental Information Service (Namibia; http://www.the-eis.com). We assumed that if a vulture was recorded as being stationary within 50 m of a transmission line or pylon it was likely to be using it as a roost or perch site. To test whether vulture perching or roosting activity was more closely associated with transmission line corridors than other features in the wider landscape the density of stationary GPS locations within a 50 m buffer each side of the transmission line network was compared with the density of stationary GPS locations in the overall home range (i.e. 99% KDE contour) for each vulture. The density of stationary GPS locations within the 50 m transmission line buffer inside each vulture’s core area (i.e. 50% KDE contour) was also compared to the density inside the total core area to identify whether stationary locations were concentrated in the vicinity of power lines inside core areas. Wilcoxon signed-rank tests were used to identify significant differences in GPS location densities at the different scales with each vulture considered as a sampling unit. The proportion of stationary GPS locations recorded within 50 m of transmission lines was compared between adult and immature vultures using a Mann-Whitney test. To assess the potential for GPS tracking data to identify possible high risk areas of vulture-power line interactions the proportion of locations of Cape vulture-power line incidents with known GPS co-ordinates (437 mortalities at 126 locations) recorded in the Central Incident Register (CIR) of the Endangered Wildlife Trust (EWT) and Eskom (the main electricity distributor in the country) between May 1996 and July 2012 [16], [18], [39] that overlapped with the vultures’ core foraging ranges was calculated.

To assess vulture use of protected areas a polygon shapefile was created comprising all IUCN category I-VI protected areas and ‘national other areas’ (i.e. protected areas uncategorized by IUCN) polygons from the 2010 and 2003 World Database on Protected Areas (WDPA) [40], [41]. Each vulture’s use (*U_i_*) of protected areas was then estimated as the proportion of stationary (< 10 km**·**h^−1^) GPS locations recorded inside the protected area polygons. The availability of protected areas (*A_i_*) to each vulture was defined as the proportion of the 99% KDE contour covered by the protected areas polygons. Wilcoxon signed-rank tests were used to identify differences between *U_i_* and *A_i_* with each vulture considered as a sampling unit. Ivlev’s electivity index (*E_i_*) was then calculated as a measure of whether protected areas were visited more frequently than expected based on their availability at the overall home range scale: *E_i_*  =  (*U_i_ – A_i_*)/(*U_i_ + A_i_*) [42]. A value of zero indicated that use of protected areas was proportional to their availability, while positive (maximum  =  +1) and negative (minimum  =  -1) values indicated greater and lesser use of protected areas than expected, respectively. Use of protected areas was also assessed in the same way at the core range scale by defining *U_i_* as the proportion of the 50% KDE contour covered by the protected areas. Adult and immature vulture use (*U_i_*) of protected areas were compared using a Mann-Whitney test. The values reported in the Results section correspond to mean ± standard deviation unless stated otherwise.

## Results

Ten Cape vultures, five adults, four immatures and one juvenile (hereafter considered as an immature) were captured and tracked using GPS-GSM tracking units for 300±178 days from November 2009 to August 2011 (Table 1). The average number of GPS locations recorded per individual was 1,052±578 with 78.35±9.47% recorded as stationary (< 10 km**·**h^−1^) (Table 1). The mean and maximum speed of all moving (≥ 10 km**·**h^−1^) locations (n = 2319) was 54.54±16.93 km·h^−1^ and 115 km·h^−1^, respectively. Mean accuracy of all GPS locations on the PDOP scale was high at 2.17±1.97 (n = 9468). Tracking units stopped transmitting data prematurely (i.e. < 1 year) for five vultures for unknown reasons. The tracking unit on an immature vulture (AG351) stopped transmitting after only 12 days and the data were excluded from the analyses. Another immature vulture (AG352) travelled north through eastern Zimbabwe before heading west to an area 40 km east of Maun, Botswana, where its tracking unit ceased transmitting data. An adult vulture (AG382) was tracked for a month to an area west of the Kruger National Park *ca.* 400 km from the capture site, where its remains were found and the tracking unit recovered. The cause of death was unconfirmed. Tracking units on two adults functioned properly for more than 8 months before data transmission ceased.

**Table 1:** Home range and distance estimates for nine Cape vultures tracked by GPS-GSM units.

				Home range estimates (km^2^)			Distance estimates (km)		
Vulture ID	Age	Tracking period (days)	GPS locations	MCP	99% KDE	50% KDE	Mean (± SD) distance between consecutive locations	Maximum distance between consecutive locations	Total distance travelled/tracking days
**AG314**	Adult	479	1,636 (1,065)	165,337	69,254	3,056	21.34±26.49	212	73
**AG329**	Adult	267	793 (746)	92,092	78,847	11,113	5.17±15.58	156	15
**AG349**	Adult	234	752 (513)	56,152	52,385	5,998	20.07±28.63	185	64
**AG355**	Adult	490	1,860 (1,341)	258,294	149,687	10,071	16.70±25.35	174	63
**AG382**	Adult	31	84 (72)	36,401	53,589	6,691	9.35±18.22	84	25
**Mean**		300	1,025	121,655	80,752	7,385	14.53	162	48
**SD**		191	722	90,845	40,095	3,250	7.00	48	26
**AG313**	Imm.	315	922 (725)	273,946	161,311	10,722	13.32±25.96	254	39
**AG352**	Imm.	207	612 (538)	392,856	312,715	44,429	9.53±20.78	159	28
**AG353**	Juv.	558	1,654 (1,251)	434,588	392,719	59,927	15.25±23.71	184	45
**AG383**	Imm.	409	1,155 (898)	867,811	737,684	80,654	13.24±21.26	192	37
**Mean**		372	1,086	492,300	401,107	48,933	12.83	197	37
**SD**		149	439	259,427	244,042	29,481	2.39	40	7

Minimum convex polygons (MCP) including all recorded GPS locations and 99% contours from kernel density estimation (KDE) represent overall foraging ranges. 50% KDE contours represent core foraging ranges. Mean (± SD) and maximum distances between consecutive GPS locations, and the total distance travelled divided by the number of tracking days are shown. The age (adult (>5 years), immature (2–5 years) or juvenile (first year)) of each vulture, the tracking period and number of GPS locations (number of stationary (<10 km·h^−1^) locations are given in parentheses) recorded are also shown.

### Size and Extent of Home Ranges

**Figure 2:**
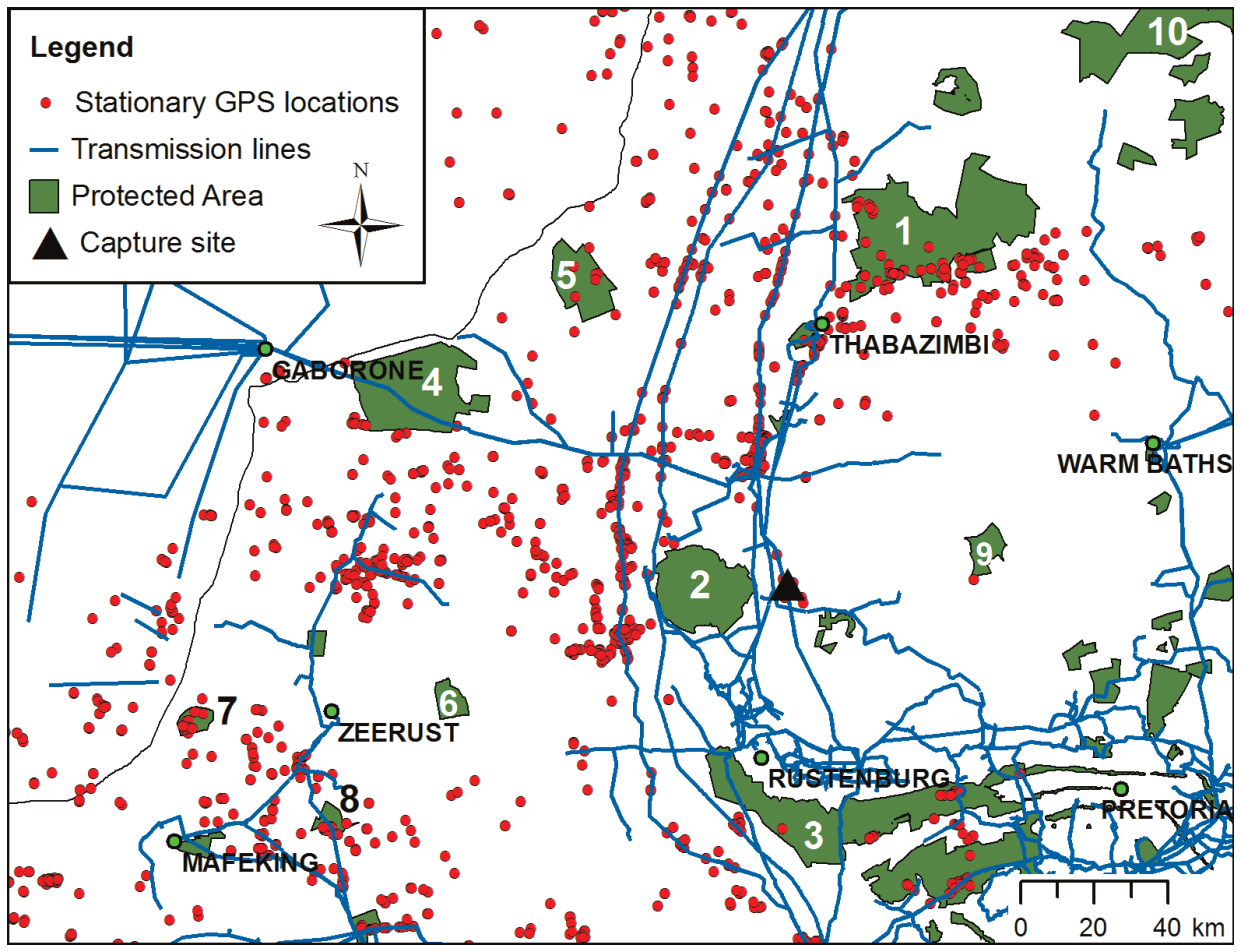
Stationary GPS locations in relation to protected areas and transmission power lines in the northern provinces of South Africa. Stationary GPS locations (red circles) from nine Cape vultures tracked by GPS-GSM tracking units are shown with transmission power lines (blue lines) and protected areas (green ploygons [40], [41]). 1  =  Marakele NP and Welgevonden NR; 2  =  Pilanesberg NP; 3  =  Magaliesberg NR; 4  =  Madikwe GR; 5  =  Atherstone NR; 6  =  Marico-Bosveld NR; 7  =  Botsalano GR; 8  =  Oog van Malmanie GR; 9  =  Borakalalo GR; 10  =  Lapalala, Moepel *et al.* reserves. The capture site is indicated by a black triangle.

The nine vultures occupied large home ranges (mean 99% KDE  =  223,132±227,256 km^2^; mean 50% KDE contour  =  25,851±28,473 km^2^; Table 1) and long distance cross-border movements were not unusual with a total of five countries (Namibia, Botswana, Zimbabwe, Lesotho and South Africa) entered by different vultures (Fig. 1A). The mean maximum distance recorded between two consecutive GPS locations for all vultures was 178±46 km (maximum  =  254 km; Table 1). Some individuals were recorded more than 1000 km straight-line distance from the capture site. Incremental area analysis indicated that the home range areas of most of the vultures had become stable by the end of their tracking periods (Fig. S1). The most intensively used areas, as indicated by the 50% KDE contours (Fig 1B), were located in the north-western Limpopo Province and north-eastern North West Provinces of South Africa, extending north into southern Botswana either side of the Limpopo River, and south to the Magaliesberg Mountains and Mafikeng district in North West Province, South Africa (Fig. 2). The 99% KDE contours (median  =  353,717 km^2^) and the MCPs (median  =  413,722 km^2^) of the four immature vultures were significantly larger than the 99% KDE contours (median  =  69,254 km^2^; *Z*  =  –2.45, *p*  =  0.014) and MCPs (median  =  92,092 km^2^; *Z* = –2.45, *p* = 0.014) of the five adult vultures (Table 1, Fig. S2).

The combined 99% KDE contours for all individuals covered 1,052,467 km^2^, of which 36% was located outside the extent of the IUCN distribution for the species, largely due to the movements of several individuals into the Northern Cape Province of South Africa and southern Namibia (Fig. 1A). A significantly higher proportion of GPS locations were recorded inside the extent of the IUCN Cape vulture distribution for adults (median  =  98.41%) compared to immatures (median  =  67.53%; *Z* = –2.21, *p* = 0.027; Fig. 1A), indicating that immatures travelled beyond the known distribution for the species more frequently than adults.

### Utilisation of Power Lines

**Figure 3:**
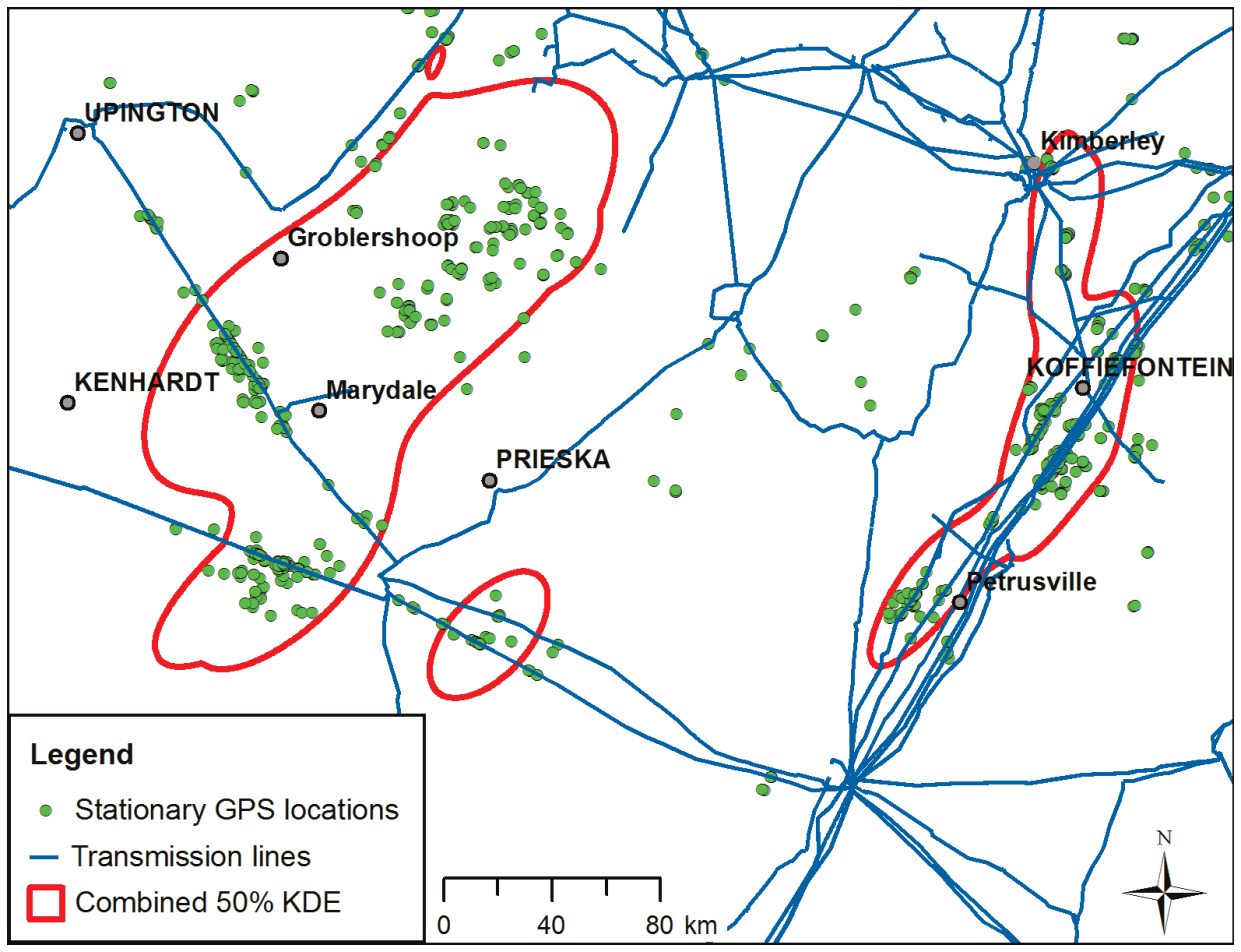
Stationary GPS locations and core areas in relation to transmission power lines in the Northern Cape Province, South Africa. Stationary GPS locations (green circles) and merged 50% kernel density estimated (KDE) contours (hollow red polygons) from nine Cape vultures are shown in relation to transmission power lines (blue lines).

The vultures were frequently recorded in the vicinity of transmission power lines. The 50 m transmission line buffer area covered only 0.52±0.14% of the 99% KDE contour areas of all nine vultures but contained 20.60±12.74% of the stationary GPS locations recorded by each tracking unit (Table S1). There was no significant difference in the proportion of stationary locations recorded within 50 m of transmission lines for adults (median  =  19.17%) compared to immatures (median  =  14.87%; *Z* = –0.490, *p* = 0.730). The density of stationary GPS locations within the 50 m transmission line buffer in the 99% KDE contours (median  =  0.267 locations·km^−2^) was significantly higher than the density in the overall 99% KDE contours (median  =  0.005 locations·km^−2^; *Z* = –2.67, *p* = 0.008; Table S1). The 50 m transmission line buffer covered significantly more of the 50% KDE contours (median  =  0.80%) than the 99% KDE contours (median  =  0.59%; *Z* = –2.37, *p* = 0.018), and the density of stationary GPS locations within the 50 m transmission line buffer in the 50% KDE contours (median  =  0.827 locations·km^−2^) was significantly higher than in the overall 50% KDE contours (median  =  0.046 locations km^−2^; *Z* = –2.67, *p* = 0.008). This indicates that the vultures were more frequently in close proximity to transmission lines when stationary compared to the wider landscape, particularly in their core foraging areas. The stationary locations within the 50 m buffer were generally clustered along certain sections of transmission line that were repeatedly visited by several different individuals (Fig. 2 and 3). Out of 126 known locations of Cape vulture-power line incidents recorded in the Eskom-EWT CIR, 120 (95%) were inside the combined 99% KDE contours of all vultures, of which 67 (56%) were recorded inside the combined 50% KDE contours (Fig. 1B).

### Utilisation of Protected Areas

All vultures spent the majority of their tracking periods outside protected areas but several regularly roosted on cliffs inside national parks or nature reserves. The difference in coverage of protected areas (Table 2) was not significantly different between the 99% (median  =  4.53%) and 50% KDE contours (median  =  9.72%; *Z* = –1.36, *p* = 0.173). Although the proportion of stationary GPS locations recorded inside protected areas (median  =  27.31%) was higher than the proportion they covered of 99% KDE contours (median  =  4.53%), the difference was not significant (*Z* = –1.84, *p* = 0.066). Ivlev’s electivity index values indicated, however, that six vultures used protected areas more than expected based on their availability in their overall home ranges (Table 2).

**Table 2:** Availability and use of protected areas by nine Cape vultures at the overall and core home range scales.

Vulture ID	PA coverage in 99% KDE (%)		PA coverage in 50% KDE (%)		Ivlev’s electivity index at core range scale		Proportion of stationary locations inside PAs (%)		Ivlev’s electivity index at home range scale
**AG314**		3.47		18.43		0.68		38.78	0.84
**AG329**		3.84		3.20		–0.09		3.49	–0.05
**AG349**		5.55		13.71		0.42		36.45	0.74
**AG355**		4.53		9.72		0.36		32.14	0.75
**AG382**		16.89		32.47		0.32		40.28	0.41
**AG313**		3.37		11.74		0.55		27.31	0.78
**AG352**		19.55		8.32		–0.40		16.54	–0.08
**AG353**		3.97		5.43		0.16		6.87	0.27
**AG383**		6.02		3.21		–0.30		4.12	–0.19
**Mean**		7.47		11.80		0.19		22.89	0.38
**SD**		6.20		9.21		0.38		15.29	0.41

The proportion of each vulture’s 99% kernel density estimated (KDE) contour occupied by protected areas (PAs) defined their availability (*A_i_*) to each vulture. At the overall home range scale use (*U_i_*) of protected areas was defined as the proportion of stationary (i.e. < 10 km**·**h^−1^) GPS locations within the 99% KDE contour that were recorded inside protected areas. The proportion of each vulture’s 50% KDE contours occupied by protected areas defined their use at the core foraging range scale. Ivlev’s electivity index values range from –1 to +1, with zero indicating use in proportion to availability, while positive and negative values indicate use more or less than expected, respectively.

Of the 1,496 stationary GPS locations recorded inside protected areas (21% of all stationary locations), 94% were in South African reserves, of which 68% were recorded in Marakele National Park (NP) in the Limpopo Province (24^o^24’S, 27^o^35’E), and 11% were recorded in the Magaliesberg Nature Reserve (NR) (25^o^44’S, 27^o^45’E), both of which encompass large Cape vulture breeding colonies (Fig. 2) [23], [26]. Marakele NP was visited by a total of seven vultures but the majority (96%) of stationary locations recorded inside the park were from three adult vultures (AG314, AG349, AG355) that frequently roosted on the breeding cliffs. 93% of stationary locations recorded inside Marakele NP were situated on the Kransberg nesting cliffs. Breeding attempts by those vultures could not be confirmed during colony observations, however, and so the influence of breeding status could not be investigated. Similarly, all stationary locations recorded in the Magaliesberg NR were situated on known breeding or roosting cliffs, the majority (87%) of which were from one immature vulture (AG313). Beyond the breeding colonies 15 other protected areas were visited in South Africa (Fig. 2), although only six contained more than 10 stationary GPS locations. Outside South Africa one immature vulture (AG383) briefly entered two protected areas in south-west Zimbabwe, another (AG353) visited the Central Kalahari GR in Botswana, while a third (AG352) entered several protected areas in eastern Zimbabwe and central Botswana. Despite the more frequent use of roost sites within protected areas by the adult vultures there was no significant difference in the proportion of stationary locations recorded within protected areas for adults (median  =  36.45%) compared to immatures (median  =  11.71%; *Z* = –1.470, *p* = 0.190).

## Discussion

This study uses GPS tracking methods to provide the first description of the relationship between the power line network and ranging behaviour of Cape vultures in southern Africa, together with their use of protected areas. The vultures, particularly immature individuals, traversed large home ranges that closely followed the spatial distribution of transmission power lines. The core foraging areas overlapped with known locations of negative vulture-power line interactions. All vultures spent the majority of their tracking periods outside protected areas, although some regularly used roost sites at breeding colonies within national parks or nature reserves.

The home ranges recorded during this study are among the largest for any vulture species. Although the five adult vultures traversed larger home ranges (mean MCP  =  121,655±90,845 km^2^) than five adult Cape vultures tracked in Namibia (mean MCP  =  21,320 km^2^ [27]), such comparisons should be considered with caution because breeding attempts by the vultures from this study could not be confirmed during colony surveys. If they were non-breeding birds their foraging movements would not have been restricted by the need to return to a nest site, allowing them to range further than breeding individuals from the Namibian study [1], [27]. The four immature vultures occupied similarly extensive home ranges (mean MCP  =  492,300±259,427 km^2^) to two immature vultures from the Namibian study (mean MCP  =  482,276 km^2^) [27]), but larger than those of six immature African white-backed vultures tracked from South Africa (mean MCP  =  269,103±197,187 km^2^ [24]). Compared to *Gyps* species outside Africa the home ranges recorded here exceeded those of Eurasian griffon vultures (*G. fulvus*) tracked in France (combined MCP  =  *c.* 1,000 km^2^ (n = 28) [43]) and Spain (median MCP  =  7,419 km^2^ (n = 8) [44]), and Asian white-backed vultures (*G. bengalensis*) in Pakistan (mean MCP  =  24,155 km^2^ (n = 6) [45]). A recent study in Israel reported that while the majority of 43 tagged *G. fulvus* did not travel more than 200 km from the centre of their home range, a few individuals undertook infrequent “long-range forays” of more than 1,700 km from their home range centres [46]. Such comparisons must be considered with caution, however, as factors that determine home range characteristics such as food availability, habitat quality, topography and levels of competition are likely to vary geographically and between species [43], and could not be fully investigated here due to limited data availability. Nevertheless, the similar long-distance cross-border movements and large distances travelled on a daily basis during this study confirm that *Gyps* vultures and Cape vultures in particular, are among the widest ranging bird species probably due to their reliance on a sparsely and unpredictably distributed food source [1], [47].

The high densities of stationary GPS locations recorded in close proximity to transmission lines provide strong evidence that the movement patterns of Cape vultures are closely linked to the spatial extent of the transmission power line network in southern Africa and suggest that they prefer to perch, roost and forage in the vicinity of transmission line towers rather than moving randomly throughout their home ranges as might be expected from a typical central-place forager [43]. For instance, although the spatial extent of the core ranges (Fig. 1B) corresponded with areas known to be important foraging grounds for *Gyps* vultures in southern Africa [23], [24], [26], [48], the core area used by three immature vultures in the Marydale region of the Northern Cape Province (Fig. 3) extended more than 100 km west of the IUCN distribution range for the species [25]. The close association of the vultures’ movements with the transmission lines in that area provides strong evidence that the construction of power line “towers have proved ideal as roosting sites.....in places devoid of cliffs”, allowing the species to expand its range into new foraging areas [4]. It is possible that the construction of power lines in that area has provided a “nursery area” where immature Cape vultures forage away from the competition imposed by dominant adult vultures at carcasses nearer breeding colonies [4], [49]. A similar finding was recorded in immature Spanish imperial eagles *Aquila adalberti* which frequently perched on pylons where alternative perching sites were limited in dispersal areas away from adult competition [50]. This could also partially explain why the immature vultures traversed more extensive home ranges than the adults, as seen elsewhere [27], [47], [49]. Although the breeding status of the adult vultures was unknown it is likely that they would have remained in closer proximity to nesting colonies in order to encounter potential breeding opportunities, compared to the immature vultures which could range further between food sources [1], [49]. However, further research is required to determine the primary factors driving the long-distance movements of immature vultures.

Although power line towers provide vultures with additional roost sites and vantage points, the large proportion of time that they spend in the vicinity of overhead cables associated with the structures, in combination with their large size, susceptibility to collisions with man-made structures and their gregarious nature puts them at significant risk [51], [52]. These factors explain the high and increasing number of collision-related injuries and fatalities of vultures recorded in South Africa [16], [17]. In some regions it is conservatively estimated that power lines kill at least 4% of the local population of Cape vultures annually [16]. The number of vultures killed by collisions is thought to be significantly under-recorded as they rarely cause electricity supply faults and are therefore not investigated, and the vast majority of vulture carcasses are likely to be removed by terrestrial scavengers before they are detected [16], [18]. If the estimate of only 2.6% of power line mortalities of blue cranes *Anthropoides paradiseus* and Denham’s bustards *Neotis denhami* being recorded in part of South Africa [53] is repeated for Cape vultures, then such a prevalent unnatural mortality factor is likely to cause severe population declines [16] as witnessed in other species [54]. For example, negative interactions with power lines are a major cause of mortality in Spanish imperial eagles, particularly in sub-adults which frequently perch on electricity pylons in areas lacking suitable alternatives [50].

Although organizations such as Eskom have invested significant resources in an attempt to reduce vulture mortalities, more widespread mitigation measures are required to prevent vulture population declines caused by the expanding power line network [16], [17]. For example, marking wires with bird flight diverters to increase their visibility and reduce the risk of collision has been carried out in many areas with some success [18], [55]. It is a costly measure (e.g. 1,100–2,600 US$ km^−1^ [56]), however, and it is therefore essential to target high risk areas. The ease of identifying repeatedly visited sections of power line and the relatively high degree of overlap between the vulture core ranges and the known fatalities recorded in the CIR recorded during this study demonstrate the ability of GPS tracking data to inform the implementation of such mitigation measures. For example, additional surveys for vulture carcasses could be carried out at frequently visited sections of power line to determine whether mitigation measures (e.g. bird flight diverters) are required or to assess their effectiveness after installation.

Although protected areas away from breeding colonies were rarely visited by any of the vultures during this study (Fig. 2), breeding cliffs inside two protected areas were regularly used as roost sites by three adults and one immature, confirming that protected areas are important for reducing anthropogenic disturbance at nest and roost sites [13], [15], [21], [22], [57]. The most intensively used areas by the vultures were located in a south-westerly direction from the Kransberg colony (Marakele NP) on private and communal farmland, and rarely included protected wildlife reserves. Although data relating to food availability were not available, this supports suggestions that Cape vultures from the Kransberg colony regularly feed on domestic livestock carcasses [58] and are therefore at risk of exposure to harmful veterinary drugs [59], [60]. The vultures also frequently travelled to the northern Limpopo Province and elsewhere in South Africa where game farming is common [61] and so it is likely that they also consumed wild ungulate species as seen previously [62]. Consequently, during their regular foraging activity the vultures would have been afforded very little protection from widespread threats such as consuming ungulate carcasses contaminated with veterinary drugs, illegal poisons used for predator control or lead bullet fragments from hunting activity on unprotected farmland [15], [61], [63]. A similar pattern of limited use of protected areas was observed for immature African white-backed vultures tracked in the same area [24]. Thus these findings further emphasise the need to establish vulture monitoring and conservation measures outside protected areas.

The small sample size of tracked vultures (n = 10) limited by financial constraints mean that the results from this study provide a first, rather than a comprehensive insight into the movement patterns of Cape vultures and their relationship with the power line network and protected areas in southern Africa. Although the findings allow preliminary comparisons between adult and immature movement patterns, future research should aim to elucidate the influence of additional individual characteristics such as breeding status and gender on Cape vulture ranging behaviour. Moreover, the effect of food availability on vulture movement patterns was not assessed during this study because of a lack of accurate data relating to ungulate densities and mortality rates. As an important factor in determining home range characteristics [33] and the risk posed by power lines at a local scale [64], this issue should be investigated further. Nonetheless, the regular sampling intervals and highly accurate GPS location data have demonstrated the ability of GPS tracking data to delineate the home ranges of vultures and assess their exposure to potential threats in the region.

## Conclusions

The findings from this study demonstrate that Cape vultures have extended their range by using transmission power line structures for roosting and perching in areas otherwise devoid of suitable perches, but must frequently face the risk of colliding with overhead wires. If the extensive movement patterns and limited use of protected areas recorded during this study are representative across the species’ geographical range then it is likely that the population is regularly exposed to multiple threats such as negative interactions with power lines and poisoning from contaminated carcasses on private farmland. We suggest that co-ordinated cross-border conservation measures beyond the boundaries of the protected area network will be necessary to ensure the future survival of threatened vultures in Africa. Specifically, additional monitoring and mitigation of negative interactions with power lines will be required, as well as a concerted effort to remove contaminants from the food supply. The use of GPS tracking data to inform conservation management of other threatened species is also advocated.

## Supporting Information

Figure S1 Home range area curves from incremental area analysis of GPS locations from nine Cape vultures**.** The number of GPS locations used to generate minimum convex polygons (MCPs) by adding consecutive locations until all locations were used is plotted against the area of each MCP. (A) – (I) represent different vultures (refer to Table 1).
(TIF)

Figure S2 Minimum convex polygons of five adult and four immature Cape vultures tracked by GPS-GSM tracking units. Hollow red and blue polygons represent merged minimum convex polygons (MCPs) using all locations from five adult and four immature Cape vultures tracked using GPS-GSM tracking units, respectively. The capture site is indicated by a black triangle.
(TIF)

Table S1 Association of GPS tracking locations and home ranges of nine Cape vultures with the transmission power line network. The proportion of the 99% and 50% kernel density estimated (KDE) contours covered by the 50 m transmission line (Tx) buffer, and the proportion of stationary GPS locations recorded within the Tx buffer are shown, as well as the corresponding stationary GPS location densities within the 99% and 50% contours and the Tx buffer.
(PDF)
