# Correction: Do Power Lines and Protected Areas Present a Catch-22 Situation for Cape Vultures (*Gyps coprotheres*)?

**DOI:** 10.1371/annotation/f4701461-4b42-4744-8338-0c15c9065800

**Published:** 2013-10-15

**Authors:** W. Louis Phipps, Kerri Wolter, Michael D. Michael, Lynne M. MacTavish, Richard W. Yarnell

This article was republished on October 14, 2013 to remove tables unrelated to this work. Please download this article again to view the correct tables. 

