# Supplementary figures and images for: Do Power Lines and Protected Areas Present a Catch-22 Situation for Cape Vultures (Gyps coprotheres)?
Source: PLoS One. 2013 Oct 9;8(10):e76794. doi: 10.1371/journal.pone.0076794 (PMC3793913; doi:10.1371/journal.pone.0076794)

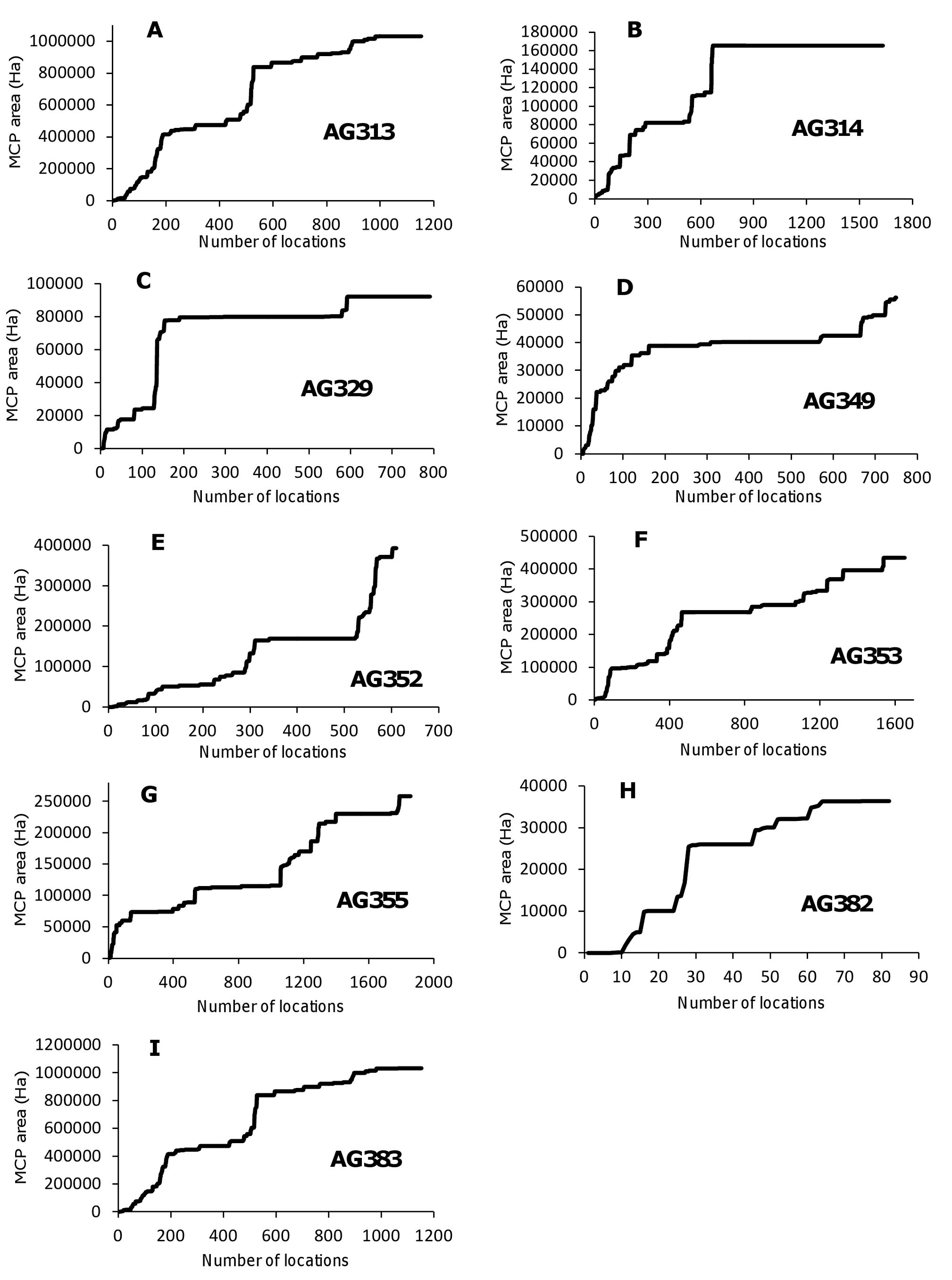

Supplement: Figure S1 — Home range area curves from incremental area analysis of GPS locations from nine Cape vultures. The number of GPS locations used to generate minimum convex polygons (MCPs) by adding consecutive locations until all locations were used is plotted against the area of each MCP. (A) – (I) represent different vultures (refer to Table 1). (TIF) [file pone.0076794.s001.tif]

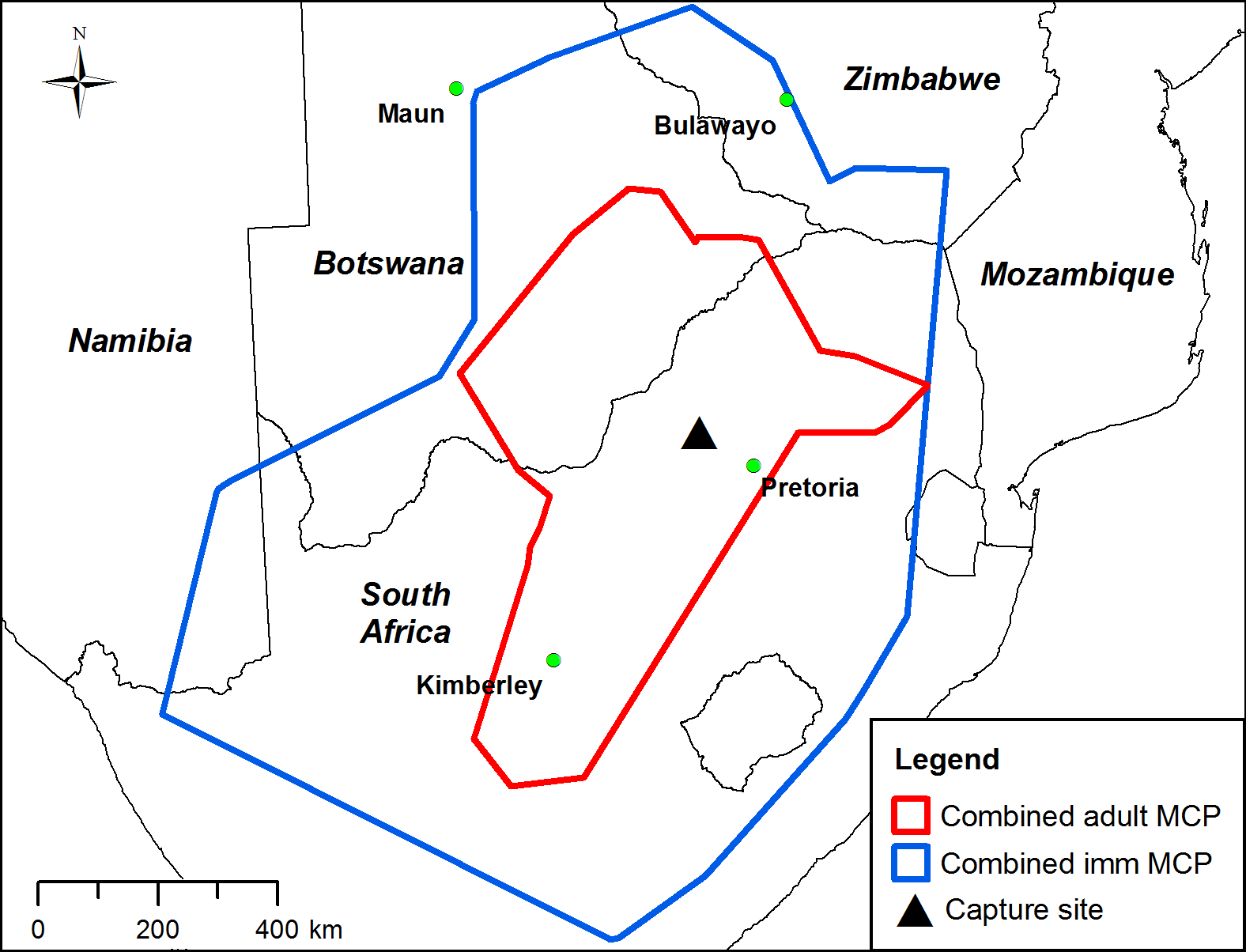

Supplement: Figure S2 — Minimum convex polygons of five adult and four immature Cape vultures tracked by GPS-GSM tracking units. Hollow red and blue polygons represent merged minimum convex polygons (MCPs) using all locations from five adult and four immature Cape vultures tracked using GPS-GSM tracking units, respectively. The capture site is indicated by a black triangle. (TIF) [file pone.0076794.s002.tif]
