# Supplementary material for: Do Power Lines and Protected Areas Present a Catch-22 Situation for Cape Vultures (Gyps coprotheres)?
Source: PLoS One. 2013 Oct 9;8(10):e76794. doi: 10.1371/journal.pone.0076794 (PMC3793913; doi:10.1371/journal.pone.0076794)
Supplement: Table S1 — Association of GPS tracking locations and home ranges of nine Cape vultures with the transmission power line network. The proportion of the 99% and 50% kernel density estimated (KDE) contours covered by the 50 m transmission line (Tx) buffer, and the proportion of stationary GPS locations recorded within the Tx buffer are shown, as well as the corresponding stationary GPS location densities within the 99% and 50% contours and the Tx buffer. (PDF) [file pone.0076794.s003.pdf]

Table S1. Association of GPS tracking locations and home ranges of nine Cape vultures with the transmission power line network.

					Stationary location density (locations·km^{-2})			
**Vulture ID**	**Proportion of 99% KDE occupied by Tx buffer (%)**	**Proportion of stationary locations in 99% KDE in Tx buffer (%)**	**Proportion of 50% KDE occupied by Tx buffer (%)**	**Proportion of stationary locations in 50% KDE in Tx buffer (%)**	**99% KDE**	**99% KDE Tx buffer**	**50% KDE**	**50% KDE Tx buffer**
**AG314**	0.39	18.31	0.98	18.70	0.015	0.730	0.222	4.233
**AG329**	0.68	19.17	1.25	20.65	0.009	0.267	0.050	0.827
**AG349**	0.59	30.99	0.80	30.60	0.010	0.513	0.067	2.563
**AG355**	0.63	32.07	1.18	32.08	0.009	0.458	0.098	2.647
**AG382**	0.66	2.78	0.51	1.56	0.001	0.006	0.010	0.029
**AG313**	0.63	43.59	0.95	46.73	0.004	0.312	0.046	2.245
**AG352**	0.35	8.74	0.61	10.71	0.002	0.043	0.009	0.156
**AG353**	0.39	14.71	0.45	17.77	0.003	0.121	0.013	0.530
**AG383**	0.37	15.03	0.52	19.04	0.001	0.050	0.008	0.294
**Mean±**	0.52	20.60	0.81	21.98	0.006	0.278	0.058	1.503
**SD**	0.14	12.74	0.30	13.09	0.005	0.250	0.069	1.470
